# New Developments in the Pathophysiology, Workup, and Diagnosis of Dural Venous Sinus Thrombosis (DVST) and a Systematic Review of Endovascular Treatments

**DOI:** 10.14336/AD.2016.0915

**Published:** 2017-04-01

**Authors:** Sanjay Konakondla, Clemens M. Schirmer, Fengwu Li, Xiaogun Geng, Yuchuan Ding

**Affiliations:** ^1^ Department of Neurosurgery, Geisinger Health System, Danville, PA 17822, USA; ^2^ Department of Neurology, Beijing Luhe Hospital, Capital Medical University, Beijing, China; ^3^ Department of Neurosurgery, Wayne State University School of Medicine, Detroit, MI 48201, USA

**Keywords:** Dural Venous Sinus Thrombosis, Cerebral Venous Sinus Thrombosis, Intrasinus Thrombolysis, Mechanical Thrombectomy

## Abstract

Dural venous sinus thrombosis (DVST) is a rare cause of stroke, which typically affects young women. The importance of identifying pre-disposing factors that lead to venous stasis lies in the foundation of understanding the etiology, pathophysiology and clinical presentation. The precise therapeutic role of interventional therapies is not fully understood though the current data do suggest potential applications. The aim of the study was to perform a systematic review and meta-analysis to evaluate the utility of and short-term 30-day survival after endovascular therapy for patients with DVST. Standard PRISMA guidelines were followed. Data sources included PubMed keywords and phrases, which were also incorporated into a MeSH search to yield articles indexed in Medline over a 5-year period. All RCTs, observational cohort studies, and administrative registries comparing or reporting DVST were included. Sixty-six studies met inclusion criteria. 35 articles investigating treatment in a summation of 10,285 patients were eligible for data extraction and included in the review of treatment modalities. A total of 312 patients were included for statistical analysis. All patients included received endovascular intervention with direct thrombolysis, mechanical thrombectomy or both. 133 (42.6%) patients were documented to have a neurologic decline, which prompted endovascular intervention. All patients who had endovascular interventions were those who were started on and failed systemic anticoagulation. 44 patients were reported to have intracranial hemorrhages after intervention. Regardless of systemic anticoagulation, patients were still reported to have complications of VTE and PE. Primary outcome at 3-6 month follow up revealed mRS<1 in 224 patients. DVST presents with many diagnostic and therapeutic challenges. The utility of invasive interventions such as local thrombolysis and mechanical thrombectomy is not fully understood. It is exceedingly difficult to conduct large randomized trials for this low incidence disease process with large pathophysiological heterogeneity.

The diagnosis of dural venous sinus thrombosis refers to an identified cerebral sinus that is obstructed by a blood clot sitting within the venous lumen. This hindrance of venous drainage leads to an increase in venous and capillary back pressure, resulting in an increase in cerebral edema, and subsequently leading to an increase in intracranial pressure and intracerebral hemorrhage (1).

The incidence of DVST has been reported to comprise of about 0.5-2% of all cerebral strokes with the precise incidence remaining unknown (1-3). Unique to DVST, younger individuals, less than 50 years of age, are affected and has an average rate of death or dependency of fifteen percent (4,5). Women are more commonly diagnosed with DVST than men (6,7). The most common sinuses affected are the superior sagittal sinus (SSS), which accounts for approximately 60%, followed by the transverse and the sigmoid sinuses, accounting for approximately 50%. Less commonly, deep cerebral venous occlusions do occur with the internal cerebral vein and the vein of Galen (11%) or the straight sinus (11%), and these patients have greater morbidity (2,6,8). Depending on the extent of cerebral injury, due to venous infarction, cerebral edema, and increased intracranial pressure, the clinical presentation may vary from asymptomatic, mild headaches, nausea, vomiting, to focal and visual deficits, stupor, coma, and death.

The general consensus that most conditions which render the body in a hypercoagulable state predispose a patient to DVST and increases DVT risk has been widely documented (2,7,9). To date, however, there remains to be specific pathophysiological mechanisms that elude this explanation as DVSTs have been diagnosed in patients without identified hypercoagulable states. Due to these issues, making the diagnosis with a targeted workup is necessary in unveiling this seemingly occult diagnosis, which often times is delayed.

A battery of tests remains available to the diagnostician’s armamentarium which range from serum markers, Computerized Tomography (CT), Magnetic Resonance Imaging (MRIs), to Digital Subtraction Angiography (DSA). Once a diagnosis is made, prompt therapeutic interventions should be considered to prevent detrimental neurologic sequelae. These interventions include, but are not limited to correction of underlying pathophysiology, systemic anticoagulation, targeted thrombolysis via interventional procedures, and close neurological checks with or without intracranial pressure monitoring. Many obstacles still remain, however, even after a diagnosis of DVST is made. For example, a patient presenting after multi-trauma with an increased risk of hemorrhage, could contraindicate anticoagulation therapy for DVST and delay treatment.

To date, the literature investigates cerebral venous sinus thrombosis (CVST) in great detail and a search including DVST yields similar results. As such, for all intents and purposes, in this review, DVST should be recognized as a subset of CVST, noting similar paradigms with both pathological descriptions.

With this review we seek to address the current state of the literature with particular emphasis to the recent interest in endovascular treatment options and remedy some of the misunderstandings that we see in daily clinical practice as to the optimal way to diagnose and manage DVST using the currently available modalities.

## MATERIALS AND METHODS

This review was conducted and subsequently reported according to the PRISMA guidelines (10). The methodological quality of observational studies was assessed using the Newcastle-Ottawa Scale. The PICOS strategy was used for describing the eligibility criteria for the selection of studies.

### Search Strategy

Using a PubMed search, three primary investigators (SK, CS, YD) reviewed relevant published articles independently within the last 5 years until October 1, 2015 with search phrases including, “Venous Sinus Thrombosis”; “Dural Venous Sinus Thrombosis”; “Cerebral Venous Sinus Thrombosis”; “Dural Venous Sinus Thrombosis Management”; “Dural Venous Sinus Treatment,” “Thrombolysis AND Dural venous sinus thrombosis,” “Thrombectomy AND Dural venous sinus thrombosis.” These terms and keywords were also incorporated into a Medical Subject Headings (MeSH) search to yield articles indexed in Medline. All RCTs, observational cohort studies, and administrative registries comparing or reporting DVST were included.

### Eligibility Criteria

Titles and abstracts of the articles yielded through the keyword searches were read and full articles were selected by relevance. The articles were deemed relevant based on the specific information provided. The articles that assisted with our purpose of relaying general information, pathophysiology/mechanisms, clinical workup, and treatment of DVST were included. A review of referenced articles in a similar fashion was also undertaken to supplement our search. Papers that were just outside of the 5-year inclusion criteria, were critically analyzed and included if relevant information, otherwise not obtained, was provided. All SR studies reporting more than once on the same patient population were included only once. Editorials, reviews, and comments were not included.

**Figure 1.**
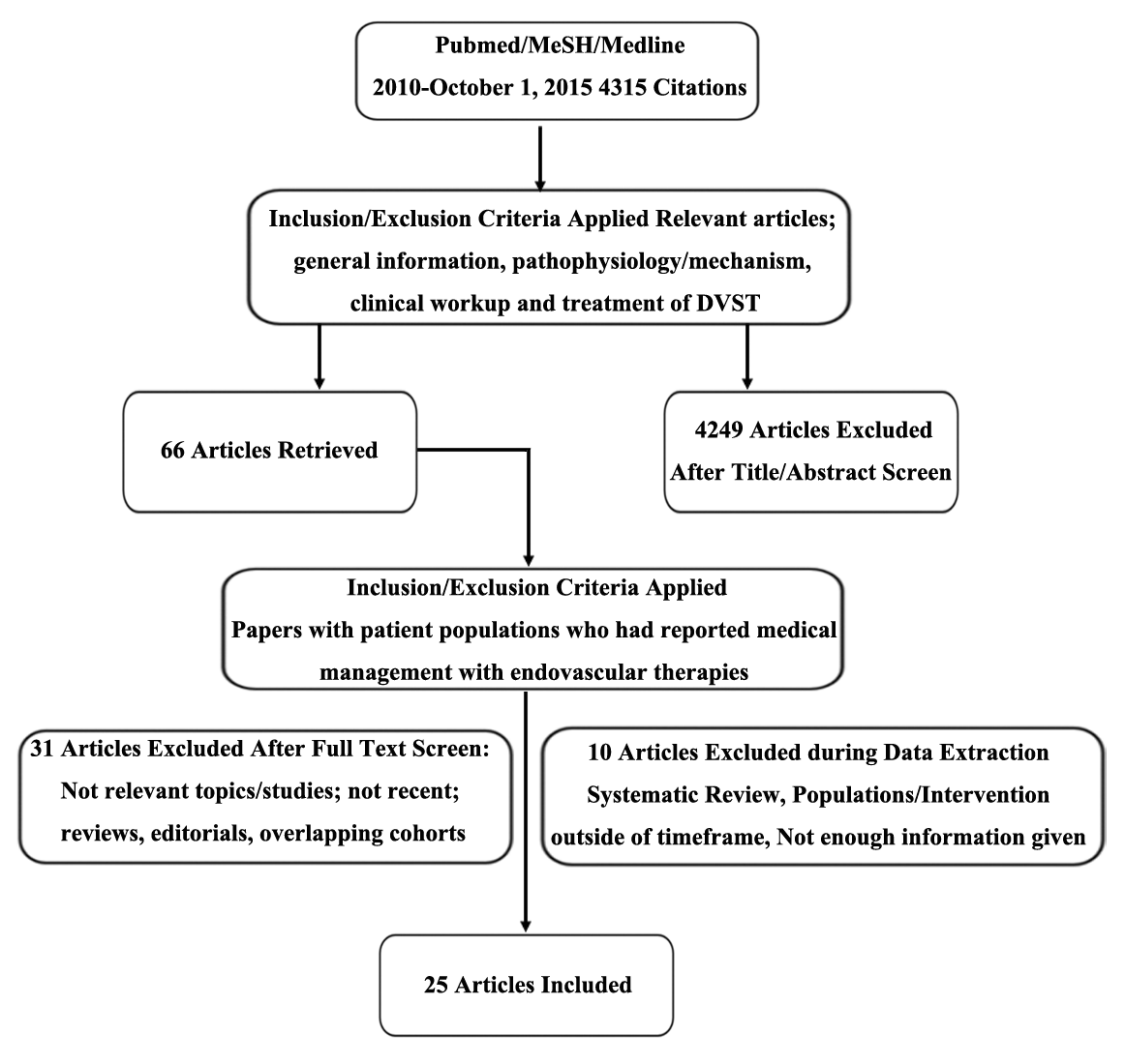
**Selection Flow chart**. Out of 4315 citations yielded in the initial search, 66 articles were retrieved (4249 excluded) after all article titles and abstracts were screened. From the 66 papers selected, 31 articles were excluded due to article type and overlapping cohorts. Ten (10) more articles were excluded during data extraction due to non-primary data, and outdated or insufficient information. Twenty-five (25) articles that reported primary data for patients receiving medical and endovascular therapies remained for statistical analysis.

A data abstraction tool was fashioned to systematically review the recent papers related to DVST treatment, medical, surgical or both. Authors, date of publication, type of study, years of data collected, patient demographics, risk factors, thrombus location, medical management, type of intervention, neurologic decline, statistical analysis, intracerebral hemorrhage (ICH), venous thromboembolisms (VTE), 30-day mortality rate, outcome measures (mRS), and follow up periods were extracted and reported. Papers that did not meet the above criteria for relevance and papers that did not discuss treatment options were excluded from the systematic review. Data gathered prior to the 5-year inclusion criteria, but reported in subsequent reviews published within 5 years were included. A selection flow chart can be seen in Figure 1.

### Statistical Analysis

The primary endpoint was the combined 30-day and in-hospital death rate. If not reported, the 30-day or in-hospital death rate was used instead. A secondary endpoint was diagnosis of new or progressively increased ICH after endovascular intervention.

## RESULTS

A total of 4315 published papers were yielded. Sixty-six studies were deemed relevant for the purpose of relaying appropriate information in the desired timeframe. From these articles, 35 articles (4,5,8,11-42) investigating treatment of a summation of 10,285 patients were eligible for data extraction and included in the systematic review of treatment modalities.

**Figure 2.**
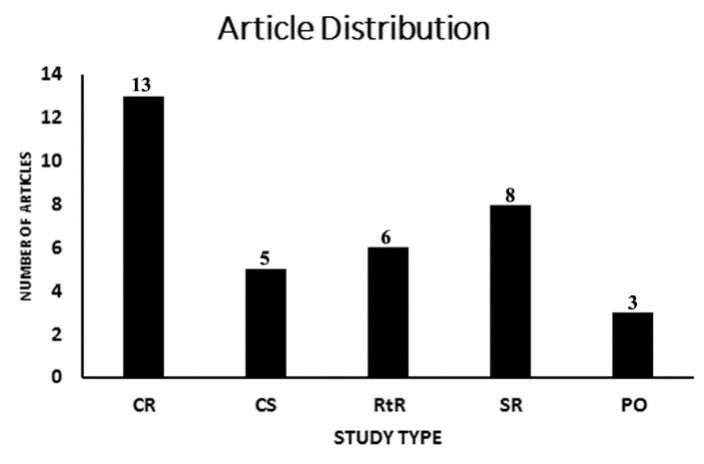
**Article distribution**. A total of thirty-five (35) published articles reporting data for patients receiving medical and endovascular therapies were selected for data abstraction. The number of articles for each study type was subcategorized: Individual case reports (CR) (13/35; 37.1%) (11,12,14,19, 21,26,28,31,33,35,37,39,41), case series (CS) (5/35; 14.2%) (13,18,20,25,40), retrospective reviews (RtR) (6/35; 17.1%) (16,22,29,30,36,38), systematic reviews of case reports/case studies (SR) (8/35; 22.8%) (4,5,8,15,17,23,24,34), and observational studies (PO) (3/35, 8.5%) (27,32,42).

Eight (8/35; 22.8%) (4,5,8,15,17,23,24,34) were systematic reviews of case reports and case series. Individual case reports (13/35; 37.1%) (11,12,14,19, 21,26,28,31,33,35,37,39,41), case series (5/35; 14.2%) (13,18,20,25,40), retrospective reviews (6/35; 17.1%) (16,22,29,30,36,38), and observational studies (3/35, 8.5%) (27,32,42) that were not otherwise mentioned or included in selected systematic reviews were included (Fig. 2). Of the retrospective reviews, 2 (2/35; 0.5%) (22,36) were multi-institutional. 10 articles were excluded as they did not meet pre-defined eligibility criteria.

A total of 312 patients from twenty-five (11,13,14,17-22,25-33,35-39,41,42) articles were included for statistical analysis. All patients included, 100%, received endovascular intervention with direct thrombolysis, mechanical thrombectomy or both. 133 (42.6%) (11,13,14,17-19,21,22,27-29,31-33,35-39,41, 42) patients reported in 21 articles were documented to have neurologic decline which prompted endovascular intervention.

**Figure 3.**
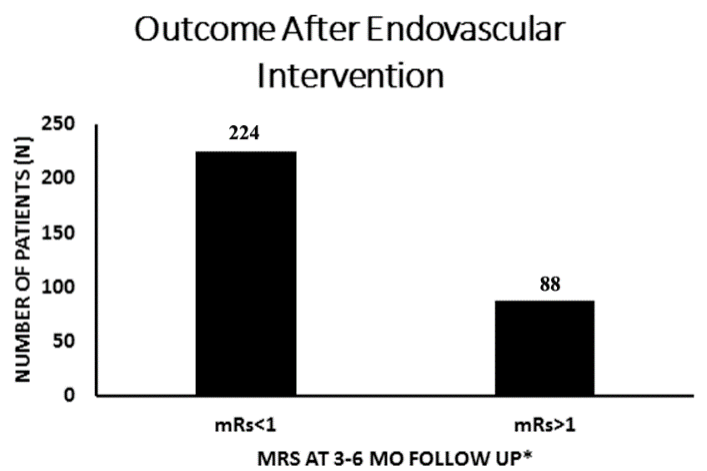
**Outcome after endovascular intervention**. Primary outcome of 30-day mortality rate ranged from 0-30% in the articles included in the data analysis. Follow up appointments ranged from 2 weeks to 5 years in articles that reported follow-ups, which included 312 patients (n=312). Outcome at 3-6 month follow up revealed mRS<1 in 224 patients. Eighty-eight (88) patients were reported to have mRS>1 after undergoing both medical and endovascular interventions. *A majority of the articles described 3-6month follow-ups.

**Figure 4.**
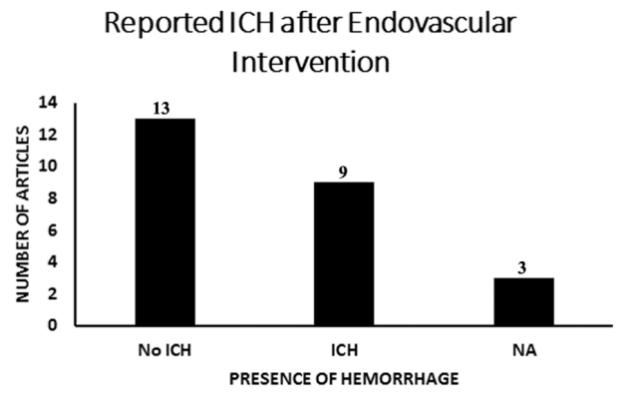
**Reported ICH after endovascular intervention**. Studies that reported the rate of intracranial hemorrhage in patients who received endovascular treatments were evaluated. Patients who had hemorrhages prior to treatment and remained stable were considered no ICH. Patients who had an increase in ICH after endovascular interventions were reported as ICH. 44 patients (14%) (22,25,27-30,38,41,42) in 9 articles at a rate of 13.2%, were reported to have intracranial hemorrhages, new or enlarging, after receiving endovascular thrombolysis or mechanical thrombectomy. Three articles did not specifically address intracranial hemorrhages post operatively (Figure 4) (21,32,35). ICH=intracranial hemorrhage, NA=not applicable/not reported, no ICH = no intracranial hemorrhage.

Regardless of systemic anticoagulation, patients were still reported to have complications of VTE and PE (4,34,41). Primary outcome of 30-day-mortality rate in individual articles ranged from 0% to 30%, with a mean of 3.8%. Follow up appointments ranged from 2 weeks to 5 years with most articles describing 3-6month follow ups. Outcome at 3-6 month follow up revealed mRS<1 in 224 patients (Fig. 3). Secondary outcome of 44 patients (14%) (22,25,27-30,38,41,42) at a rate of 13.2%, were reported to have intracranial hemorrhages, new or enlarging, after receiving endovascular thrombolysis or mechanical thrombectomy. Three articles did not specifically address intracranial hemorrhages post operatively (Fig. 4) (21,32,35).

All patients who had endovascular interventions were those who were started on and failed systemic anticoagulation. Four patients, in total, were reported to receive decompressive craniectomies. Three of these patients had reported favorable outcomes. Regardless of systemic anticoagulation, patients were still reported to have complications of venous thromboembolism (VTE) and pulmonary embolism (PE). This was attributed to the already existing prothrombotic risk factors. A summary of recent and relevant studies investigating intervention, whether they were medical, surgical, or both, are shown in Table 1.

**Table 1:** Articles describing endovascular interventions

Author (year)	Years Studied	Study Type	n=x	AC/TL	Intervention	STATS	Outcome (f/u)	ICH	VTE
**Shaikh (2015)(12)**	2015	CR	1	Heparin	Solitaire FR + Penumbra		NA	NA	NA
**Pan (2015)(11)**	2014	CR	1	AG	IST (UK) + MT		6mo	0	NA
**Liao (2015)(13)**	2014	CR	2	Heparin	IST (UK) + MT		mRS <1	0	NA
**El Tecle (2015)(14)**	2014	CR	1	Heparin	IST+MT Penumbra		mRS<1 (6mo)	NA	NA
**Siddiqui (2015)(15)**	1995-2014	SR	185		MT+/- IST	Medians, odd ratios, outcomes	Mortality mRS<3=good (84%)	YES^*^ (60%)	
**Arauz (2014)(16)**	2000-2013	RtR	102	Heparin	NA		mRS<1 (3mo)	0	0
**Zhen (2014)(17)**	2009-2011	SR	8	Heparin	IST (tPA)+ MT		mRS<1 (3mo)	0	0
**Shui (2014)(18)**	2006-2012	CS	26	NA	MT (balloon dilation)		mRS<1 (12-62mo)	0	0
**Pukenas (2014)(19)**	2014	CR	1	Anticoag	AngioJet+ Solitair FR		mRS<1 (NA)	0	0
**Guo (2014)(20)**	2013	CS	2	NA	IST (UK)		mRS<1 (6mo)	0	0
**Raychev (2014)(21)**	2013	CR	1	IV Heparin	MT	NA		NA	NA
**Siddiqui (2014) (22)**	1995-2012	MI RtR	63	Heparin	29 IST- (23UK, 6rtPA) 34 MT(+/- IST)	Fisher exact, Wilcoxon rank, Cox/logistic regression	Mortality neuro status, 3-6mo mRS<1 = good^***^	YES	
**Coutinho (2014) (23)**	1942-2012	SR	8829	71.8% Heparin (UFH, LMWH)	Thrombolysis 9.2%; Craniectomy 3.6%	Pearson Correlation, Sensitivity Analysis	Mortality (median 14mo)	NA	NA
**Haghighi (2014)(24)**	1990-2012	SR	64	60 (93.8%) IV Heparin	49 MT,	Chi Square, Fisher exact	40mRS 0-2 (Mean 28.3 wks)	YES 31^+^	
					12 BV,				
					3 BVS,				
					41 IST				
**Viegas (2014)(8)**	Up to 2011	SR	26	19 (73.1%) UK, 2(7.7%) SK, 2(7.7%) rtPA	NA	Survival rate	mRS	YES 3 1.5%^++^	NA
**Gala (2013)(5)**	2001-2008	SR	59	NA	23 UK, 2 rtPA		82% MT “good outcome”	YES^**^	
					1 UK+AP				
					1 rtPA+AP				
					32 MT				
**Yakovlev (2014)(25)**	2005-2012	CS	16	NA	IST(15)+MT (4)		mRS (1-5 yrs)	1	NA
**Rammos (2013)(26)**	2012	CR	1	Heparin	MT, IST (tPA)		mRS (1yr)	0	NA
**Li (2013)(27)**	2007 to 2010	POS	52	IV Heparin	MT + IST (UK)	Student t test, x^2^	mRS/GCS (3+6mo)	YES 35	
**Poulsen (2013)(28)**	2007-2011	CR	9	Heparin	IST rtPA +/- MT(6)		mRS(6mo-4yrs)	YES	
**Mortimer (2013)(29)**	1999-2013	RrT	9	Heparin(8/9)	IST (8/9); MT		mRS (NA)	2	NA
**Guo (2012)(30)**	2007-2009	RtR	37	Heparin, Coumadin	IST (UK)	NA	mRS(6mo)	YES, 2	NA
**Velat (2012)(31)**	2012	CR	1	Heparin	MT Penumbra		mRS (6mo)	0	NA
**Mohammadian (2012)(32)**	2008-2011	POS	26	Heparin	IST (rt-PA)		mRS (3 mo - 23 mo)	NA	NA
**Froehler (2012)(33)**	2012	CR	1	Heparin	IST (rt-PA) + MT Solitaire FR		mRS (3mo)	0	NA
**Coutinho (2012)(34)**	1991, 1999	SR	79	10UF, 30 LMWH	NA	RR, ARR	RR0.46 ARR 13%	NO	YES (PE in Placebo group)
**Shah (2011)(35)**	2011	CR	1	Coumadin	IST (tPA) + MT		NA		NA
**Dashti (2011)(36)**	2009-2010	MI RtR	13	Heparin	MT (AngioJet)		mRS (2wks-9 mo (5mo mean)		NA
**Blackham (2011)(37)**	2011	CR	1	Heparin	IST (rt-PA) + MT Penumbra		mRS (3 wks)		NA
**Kumar (2010)(38)**	2003-2004	RtR	19	Heparin	IST (UK) +/- MT		6 mo - 8 mo	13	NA
**Atherton (2010)(39)**	2010	CR	1	NA	IST (rt-PA) + MT balloon		8mo		NA
**Miranda (2010)(4)**	1998-2001	SR	624	83.3% (520) IV Heparin, LMWH	NA	KM, COX HR	VTE (median 13.9 mo)	NA	YES 36 (5.8%)^+++^
**Coutinho (2009)(40)**	2006	CS	3	Nadroparin	Hemicraniectomy		mRS (6+12mo)	YES^○^	NA
**Khan (2009)(41)**	2009	CR	1	NA	MT, BV, rtPA	NA	GCS	YES	YES^○○^
**Stam (2008)(42)**	NA	PCS	20	Heparin	IST (UK), 15MT +IST(UK)	Mann-Whitney test, Fisher exact	mRS 12<3	YES 5	

The author, year, study type, population, medical therapy, type of intervention, statistical analysis used, outcome (mRS) and follow-up time period, ICH after intervention, and VTE after anticoagulation is represented here. Abbreviations: AC, anticoagulation used; AP, angioplasty; ARR, absolute risk reduction; BV, balloon venoplasty; BVS, balloon venoplasty with stenting; COX HR, Cox hazard ratios; CR, Case Report; ECH, extracranial hemorrhage; CS, Case Series; f/u, follow up; GIH, gastrointestinal hemorrhage; ICH, intracranial hemorrhage after intervention; IST, intrasinus thrombosis; IV, intravenous; KM, Kaplan-Meier; LMWH, low molecular weight heparin; MI, multi-institutional; mRS, modified Rankin Score; MT, mechanical thrombectomy; n=*x*, population studied; NA, not available; PE, pulmonary embolism; PCS, prospective case series; POS, prospective observational study; PRC, procedure related complications; RR; relative risk; RtR, retrospective review; rtPA, recombinant tissue plasminogen activator; SK, streptokinase; SR, systematic review; STATS, statistics; TL, thrombolysis; UFH, unfractionated heparin; UK, urokinase; VTE, venous thromboembolism.

* all pretreatment ICH;

** 12% death reported in patient over 80 and with PRC;

*** IST group had minor deficits and MT group has more reported complications.

+ ICH, 14 herniation prior to MT;

++ ECH 5 (19.2%);

+++ Thrombotic events in continued AC group also.

○ mRS of 2/3 patients was 1 at 12 months;

○○ Required craniectomy; DVT in left common femoral vein; PRC.

## DISCUSSION

### Proposed Pathophysiology and Flow Dynamics

The causes for DVST have been echoed in literature to be in relation to changes in venous rheology, vessel walls, and viscosity, ultimately suggesting the predisposing cause of a hypercoagulable state. This description of Virchow’s Triad has been demonstrated by various studies and observed etiologies have been centered on this basis (1-3,8). The specific pathogenesis of cerebral venous sinus thrombosis, however still remains poorly understood as animal models have been unsuccessful in reproducing models reasonably representative of humans (43). Li and colleagues introduced a new thrombosis model of the SSS to investigate the underlying mechanisms and subsequent consequences of venous sinus thromboses. A total of 49 rats were dichotomized into two groups, experimental and sham, where the rats in the experimental group were subjected to SSS and cortical venous thromboses. Five rats died from intracranial hemorrhage giving the mortality rate of the study group 15.6%. Pathophysiologic variables including mean arterial blood pressure and temperature did not differ between the groups. Neurologic evaluations proved to yield significantly worse scores for experimental rats compared to control rats. MRI and histologic observation demonstrated strokes, cerebral edema, and hemorrhage in the brains of experimental rats, all of which were objective findings most representative of humans.

A majority of the associated diseases and predisposing risk factors for hypercoagulable states mentioned through this literature review are pregnancy, oral contraceptives, cancer/malignancies, dehydration, infections (sinusitis), thrombophilia, and other coagulopathies (Factor V Leiden, Protein C&S deficiencies, and antithrombin III deficiency) (2,7,44). In the International Study on Cerebral Vein and Dural Sinus Thrombosis (ISCVT), the female sex was stated to be one of the major predisposing risk factors and many of the identified causes of DVST were related to risk factors unique to women (7,45). There is still a certain percentage of DVSTs that are diagnosed without an identified risk factor (8). Once a diagnosis of DVST is made, an investigation of these predisposing factors should ensue. 44% of patients in the ISCVT were found to have more than one prothrombotic risk factor (7).

Mechanical causes of sinus thrombosis have been identified including direct injury to the sinus or jugular veins (ie: intraoperative sinus manipulation or jugular catheterizations) (45). Infections of areas close to venous sinuses were described to be a more common cause of sinus thromboses in the past, however with the ease of access to antimicrobials and early detection of infections, these have been much more uncommon (44-46).

After the occlusion of a venous sinus, there is an increase in intravenous pressure, the arterial to venous gradient decreases, leading to a decreased cerebral blood flow and brain tissue oxygenation. Cytotoxic edema supervenes followed by venous infarction, congestive hemorrhagic conversion and increased intracranial pressure (1,43,47).

#### Cerebral Spinal Fluid (CSF) Flow Dynamics in DVST

There have been many models demonstrating the relationship between cerebral blood flow (CBF), and CSF production, absorption and circulation. These concepts together describe the flow of CSF from the choroid plexus, where it is produced, through the ventricles, into the subarachnoid space, draining into arachnoid granulations and subsequently into cerebral veins down a CSF-venous sinus gradient. Recent studies have also elucidated the critical role of a cerebral capillary network which absorbs most of the CSF (1,45).

Thrombosis of a major sinus may affect CSF drainage indirectly as arachnoid granulations drain into the sinus and are considered to be one of the main sites for CSF absorption (45). A study showed that hydrocephalus was present in approximately 15% of patients and was associated with a higher risk of mortality (6). In contrast, Park and colleagues postulated ventricle size may decrease as they get compressed. In the setting of a thrombus within a venous sinus that does not normally play a large role in CSF drainage, ventricles may appear smaller due to the surrounding increased cerebral edema and intracranial pressure (1). With these principles in mind, the management of increased ICP and hydrocephalus in the setting of DVST proves to be challenging.

### Clinical Presentation and Progression

Impaired venous drainage with a resultant increase in intracranial pressure account for varying degrees of clinical presentations. The incomplete thrombosis or the presence of adequate collateral venous circulation allows for venous drainage to continue to some extent. Plausible presentations, therefore may range from absent clinical signs and symptoms, to vague or severe signs and symptoms related to acute increases in intracranial pressure (1,2). Headache is the most common presenting symptom by far accounting for approximately 90% of patients diagnosed with DVST, followed by seizures (6,48,49).

Local cerebral injury associated with focal ischemic changes or intracerebral hemorrhage also lead to pertinent clinical presentations and continued clinical disease progression. Approximately 50% of patients with cortical cerebral venous thrombosis develop venous infarcts (7). Therefore, focal neurologic deficit should raise suspicion for local venous infarct with the diagnosis of DVST. Depending on to the adequacy of venous drainage, timing, and associated local injury symptoms may include headaches, nausea, vomiting, dizziness, headaches, decreased visual acuity, papilledema, paralysis, altered mental status, depressed mental status, seizures, coma, death (1,8,50,51).

The number of reported cases of patients presenting with a subarachnoid hemorrhage (SAH) due to a cerebral venous sinus thrombosis is increasing and should be recognized as a possible etiology during workup (52,53). Therefore, as arterial etiologies are ruled out, venous imaging must be considered to further investigate the possibility of cerebral venous sinus thrombosis. Though the specific pathogenesis is poorly understood, one theory reasons elevated pressures within weak cortical veins resulting in focal venous rupture within the subarachnoid spaces (53). The presence of blood within the ventricles with minimal to no SAH leads others to theorize a more non-focal etiology.

### Making the Diagnosis

The undistinguished presentation of patients with DVST complicates the diagnostic workup and is, for this reason, understood to be an overlooked diagnosis requiring a highly educated clinical suspicion. The wide differential diagnoses with varying subjective and objective findings at times can suggest an alternative primary diagnosis which may not warrant further workup for an underlying DVST. Thus, with the knowledge of the previously stated potential clinical scenarios, a diagnostic endeavor must be undertaken.

Various diagnostic measures, both independently and in concert, have been completed to yield the diagnosis of DVST. These diagnostic modalities include the serum laboratory investigation, MRI/MRV, CT/CTV, cerebral angiography (8). With laboratory and imaging studies alike, the detection and appearance of a thrombus is directly linked to the time of clot formation and the time the study is performed (54).

#### Laboratory Investigation

D-dimer has been demonstrated to be notably elevated in patients suffering from extensive acute DVST. It is a marker of fibrin degradation and has been reported to be increased to levels more than 500ug/L. With this in mind, however, a low D-dimer does not rule out the presence of a DVST or a subacute to chronic DVST. A high clinical suspicion warrants further investigation even with low to normal D-dimer levels as it can also be falsely negative in patients with a high clot burden (6,46).

At the time of the clinical episode numerous abnormalities of the coagulation system can be seen which are likely resultant and not causative. While a thorough evaluation should be undertaken, a delay of 3 months after recovery from the acute phase is helpful before evaluating predisposing conditions (CBC, Factor II level, serum homocysteine level, paroxysmal nocturnal hemoglobinuria (PNH) panel, leukocyte alkaline phosphatase). Some tests investigating an underlying thrombophilia can be undertaken at any time, e.g. protein C and S levels, antiphospholipid antibodies.

#### Computerized Tomography and Computerized Tomographic Venography (CT and CTV)

A venous sinus thrombosis on a non-contrasted CT scan is represented by a hyperdensity along the vein of interest, known at the “cord sign.” This may be present for up to 2 weeks (54). Secondary effects including intracerebral hemorrhage or cerebral edema, particularly not representative of an arterial territory could suggest a cerebral venous sinus thrombosis. A triangular hyperdensity noted in the posterior third of the sagittal sinus or proximal transverse sinus known as the “delta sign,” represents a thrombosis in this area (2,9,55). A CTV with 3-D reconstructions, if preferred, may be helpful in revealing a thrombus in a major draining vein (6). Limitations of the CT include the ionizing radiation, lack of detail in regards to brain parenchyma, deep veins and smaller veins, and risks with contrast administration for CTV. These factors should be considered, especially in patients of the pediatric population (56).

#### Magnetic Resonance Imaging and Magnetic Resonance Venogram (MRI and MRV)

The MRI, due to the specific sequences available, is more sensitive than the CT and is the preferred non-invasive method of identifying a cortical or a deep venous sinus thrombus. The appearance of a thrombus on an MRI, again, depends on the age of the clot. Within the first few days a clot appears iso-intense of T1 weighted imaging and hypo-intense on T2 weighted imaging, owed to the presence of de-oxyhemoglobin (9,54,57). After approximately 1 week, the presence of methemoglobin gives the clot a hyper-intense appearance on all sequences. Contrast enhanced MRV is more sensitive than time- of-flight (TOF) MRV and can identify a thrombus in a vein of much smaller caliber (58). TOF imaging modalities are susceptible to artifact and often exaggerate signal loss especially in slow flow veins (54,59).

#### Cerebral Angiography

Cerebral angiography can be safely an easily executed when the diagnosis by MRI/MRV is uncertain, if the patient is unable to have an MRI, or if appropriate technologies are not easily accessible.

The secondary effects of hemorrhage and cerebral ischemia are also important to identify on all imaging studies with the MRI being most sensitive. Areas of particular interest could be suggested by the patient’s clinical presentation. In cases of thrombosis of deep venous networks, particularly internal cerebral veins, bilateral infarcts or hemorrhages may be identified. With local infections such as sinusitis, or otitis, or with more diffuse processes like meningitis, specific focus should be given towards areas such as the transverse or sigmoid sinuses (6,44,54).

### Treatment

#### General Management

The acute management of DVST, requires a calculated balance between three desired goals: 1) recanalization of thrombosed vessels 2) preventing medical complications of therapy and 3) prevention and treatment of secondary effects.

Though the diagnosis may be uncovered through multiple scenarios with appropriate workup, the current abundance of literature on DVST supports a quick diagnosis and treatment with fluids and anticoagulant therapy shortly after (1,2,6,8,52,53,60). The goals of anticoagulation aim to recanalize the vessel, prevent thrombus growth and prevent other thrombotic events (6). Recanalization of these vessels re-establishes venous drainage and may also help with acquired hydrocephalus. Many studies have reported anticoagulation with heparin to be safe with improvement in outcome. Consideration should be given as to the underlying condition and its correction. Infection and endocarditis can lead to infected thrombophlebitis and need to be treated with antibiotics.

#### Anticoagulation

Intravenous heparin or low molecular weight heparin (LMWH), described by ISCVT and previous randomized clinical trials, are options for acute DVST treatment (5,7). The underlying cause of DVST and associated signs and symptoms may raise question on timing of initiating this therapy. Complex clinical scenarios may preclude the immediate commencement of anticoagulant therapy. For example, patients requiring or following operative intervention, trauma, or those who present with intracerebral hemorrhages all raise concerns about early anticoagulation therapy (3,7,8,52,53,60,61). Heparin, however, has been shown to be beneficial rather than harmful for patients with DVST in the setting of ICH, although there has not been any new data or evidence since the initial finding (62). A systematic review of two randomized control trials with a total of 79 patients demonstrated safe heparin administration with no reports of symptomatic intracerebral hemorrhages in the setting of diagnosed cerebral venous thromboses (Table 1) (34). Anticoagulation therapy was associated with favorable outcome when started four days after initial onset with imaging studies revealing regression of subarachnoid hemorrhage (60).

#### Thrombolysis and Endovascular Intervention

Veigas and colleagues reported a potential beneficial role of systemic thrombolysis in a systematic review of 16 reports and 26 patients, investigating the use of systemic thrombolysis in the setting of CVST. No randomized clinical trial was found. Thrombolytic therapy varied in studies with a range of 1-120 days after symptom onset. A majority of the patients, 92.3%, regained independency (mRS<3) at last follow up. Three patients had ICH, 2 deaths associated with ICH. Current reports are not sufficient to state an obvious benefit from systemic thrombolysis versus systemic heparin (45). In an observational study of 27 patients, Guo et al demonstrated utility of early endovascular thrombolysis with clot lysis with mRS of 25 patients 0-1 at hospital discharge and 95% of patients asymptomatic or with minor deficit at 6 month follow up (30). According to the AHA/ASA there is insufficient evidence to support thrombolysis and endovascular thrombolysis may be considered in expert centers (2).

In a retrospective review of 63 non-randomized patients, Siddiqui and colleagues compared the mortality, morbidity, angiographic recanalization, and periprocedural complications between patients who received mechanical thrombectomy (MT) +/- intrasinus thrombolysis (IST) vs IST alone (Table 1) (22). Patients who received MT were in poor clinical condition prior to and after intervention and were reserved for patients after failed anticoagulation therapy. The precise definition of failed anticoagulation therapy is lacking and clinical presentation should be taken into account (22). Khan et. al described a case of successful mechanical thrombectomy performed on an obese 35-year-old female on oral contraceptives with multiple DVSTs (41). This favorable outcome, however also illustrated a missed diagnosis after CT imaging, and was accomplished after multiple endovascular interventions, intrasinus thrombolysis with rtPA, intracerebral hemorrhage, VTE/PE, procedure related complications and a craniectomy (41). Stam et al conducted a study that emphasized this observation that aggressive thrombectomy and thrombolysis may be conducted on those with severe CVST effectively and though it may produce improved clinical outcome and radiographic recanalization, it has a high risk of intracerebral hemorrhage (42). Similarly, in a retrospective review Kumar and colleagues suggested a safe role for IST in those who failed to respond to conventional medical treatment (38). In a systematic review incorporating 185 patients who received MT, new or increased ICH was the major peri-procedural complication (15). There appears to be significant reporting bias in these small series consistent with their lack of prospective data collection.

The safety and efficacy of endovascular local thrombolysis and thrombectomy was investigated by Li et al through a prospective observational study (27). Complete recanalization was observed in 45 of 52 patients who underwent MT. MT was found to significantly improve GCS and mRS when comparing pre-treatment to post-treatment patients. There were no intracranial hemorrhages in patients who did not initially present with an ICH, though 4 patients did have an increased in ICH size after intervention demonstrated by CT. A relatively low, 11.5% mortality rate, and a rapid recanalization rate of 205 minutes using dual treatment of thrombolysis and thrombectomy was demonstrated supporting this application as a safe and feasible option (27).

Though studies describing the definitive role of interventional therapies are yet to surface, endovascular options may be explored in patients with progressive neurologic decline despite aggressive medical management. Targeted thrombolysis and mechanical thrombectomy both serve as potential treatment options to those patients not adequately treated with systemic anticoagulation (21,22,28,63). Thus, specific indications, patient selection and patient safety still must be investigated with large randomized trials prior to stating recommendations for these interventions (5,24,64).

The Thrombolysis or anticoagulation for cerebral venous thrombosis (TO-ACT) trial is currently enrolling and designed to investigate the functional outcome improvement in patients with severe CVST who undergo endovascular thrombolysis (65).

#### Open Surgical Management

Though direct surgical management has been entertained and anecdotally described in the past, none of the published literature analyzed contains recommendations for direct surgical management.

#### Seizure Management

The treatment of secondary effects of DVST plays a large role in the care of the DVST patient. Seizures may occur due to intraparenchymal hemorrhages, venous infarctions, or increased ICPs and current guidelines recommend early seizure management in patients who have already had at least one seizure (2,7,22,45). They can occur in up to 40% of patient with DVST and can sometimes lead to status epilepticus (45). Routine prophylactic anti-epileptic medications have not been shown to have any benefit.

#### Intracranial pressure Monitoring

Intracranial pressure monitoring and treatment of hydrocephalus may be warranted in a patient with a poor, unreliable neurologic status, especially those with other observable intracranial abnormalities (ie: SAH) (52). Conversely, too much CSF drainage has been described to accelerate the sinus thrombosis and aggravate the patient’s symptoms, particularly with superior sagittal sinus thrombosis. The idea that aggressive CSF drainage leads to decreased venous blood flow, thus worsening the sinus thrombosis, has been proposed as a likely mechanism (1). Recommendations for re-imaging after the initiation of CSF drainage to follow for interval change of a DVST are yet to surface. Medications like acetazolamide, which decrease CSF production may also be an option. Ophthalmological consultation to follow vision changes related to papilledema is generally recommended (6). In the setting of progressive intracranial pressure, not controlled with aggressive medical management and external cerebrospinal fluid drainage, decompressive craniectomy can be considered as the next surgical step. Though the data on decompressive craniectomies for patients with DVST in the setting of uncontrolled ICPs is lacking, the current data published suggest poor outcome for patients who were comatose before and after surgery and those with bilateral lesions (64). Coutinho et al did show utility in large craniectomies performed in 3 patients with DVST and rapidly declining neurologic exams who were not responding to anticoagulation. Two out of the three patients in this case review improved to a mRS of 1 at twelve month follow up (40).

#### Outcome

According to the International Study on Cerebral Vein and Dural Sinus Thrombosis (ISCVT), outcomes are generally favorable with approximately 81% of women with complete recovery and 71% of men with complete recovery (7,51). Without continued anticoagulation, patients are at greater risk of developing other thromboembolic events in the future. It was noted to be up to 6.5% per year (6,7).

In the same study, risk factors for poor outcome were reported as male sex, age over 37 years old, mental status disorder, intracranial hemorrhage on admission, a deep venous thrombosis, CNS infection, and cancer. The rate of death and dependence was 13% compared other earlier studies that reported a death and dependence rate of 9%-44%. Seizures and new thrombotic events were the most frequent complications. Recurrence of DVST and visual loss were also noted, however noted to be preventable events (7).

Recommendations on the duration of treatment are noted to be individualized. This decision is based on risk of recurrence, patient preference, and patient risk of hemorrhage. Generally, 3-6 months of warfarin is recommended for patients with transient risk factors. Patients with initial events with mild chronic conditions may be treated for up to a year. Reports of the recurrence rate after CVST of 2% to 3% and approximately 5% for any VTE have been published (4,7). Using the ISCVT data, Miranda et al. analyzed the VTE and recurrent CVT rates and reported a 2.6% likelihood of VTEs 6 months following diagnosis. An association between anticoagulation duration and prevention of thrombotic recurrence was unable to be elucidated as 58.3% and 64.3% of patient on anticoagulation had thrombotic recurrence and recurrent CVTs respectively (4). The differences in therapeutic anticoagulant ranges and prothrombotic risk factors between patients were not evaluated and could explain these findings. Patients with ongoing genetic predisposing factors or with recurrent DVSTs may be treated indefinitely with anticoagulation (6,47). Saposnik et al, on behalf of the AHA/ASA, released a statement that outlined recommendations for anticoagulation therapy.

Using the ISCVT data, an ordinal score ranging from 0-9 was developed by Ferro and colleagues to predict the outcome of patients with CVST and DVSTs (66). This score factors in neurologic exam, intracranial hemorrhage, presence of malignancy, and involvement of the deep venous system. Higher scores predict worse outcomes as measured by the Modified Rankin Scale (mRS). The utility was proposed to decrease the use of unnecessary or dangerous interventions in low risk individuals and assist in identifying high risk patients to subsequently consider more aggressive therapies (66). In a large systematic review of 74 included publications accounting for 8829 patients, over 70 years, Coutinho and colleagues demonstrated an inverse correlation between mortality and year of patient recruitment. Prior to 2000, the year of recruitment had a significant inverse relationship to mortality (<0.001) and after 2000, publications with high mortality rates were found to have more critically ill patients included (23).

### Conclusion

DVST is a recognized cause of stroke, more common in women, and presents with many diagnostic and therapeutic challenges. Identifying predisposing risk factors and considering DVST in the differential is critical for timely therapeutic intervention and appropriate long term follow up. Early anticoagulant therapy with heparin or LMWH is indicated acutely, and the utility of invasive interventions such as local thrombolysis and mechanical thrombectomy is not fully understood. It is exceedingly difficult to conduct large randomized trials for a low incidence disease process with large pathophysiological heterogeneity as seen in DVST. While efforts such as TO-ACT are underway, it remains to be seen whether the trial is adequately powered and will be able, upon completion, to reach significance should it exist. Moreover, the rapid and recent advancements in perfecting endovascular techniques may render previously published data against intervention obsolete.
